# Imaging in plasma cell disorders—consensus recommendations of the Asian myeloma network bone imaging workgroup

**DOI:** 10.1016/j.lanwpc.2025.101597

**Published:** 2025-06-07

**Authors:** Chandramouli Nagarajan, Natasha Fay Anthony, Jens Hillengass, Seok Jin Kim, Chutima Kunacheewa, Jay T. Datukan, Gin Gin Gan, Vo Thi Thanh Binh, James Chim, Jeffery S.Y. Huang, Wenming Chen, Kihyun Kim, S. Vincent Rajkumar, Brian G.M. Durie, Wee Joo Chng, Masahiro Abe

**Affiliations:** a Department of Haematology, Singapore General Hospital, Singapore; b Department of Haematology, National Cancer Centre, Singapore; c Myeloma and Amyloidosis Service, Roswell Park Comprehensive Cancer Center, Buffalo, NY, USA; d Division of Hematology-Oncology, Department of Medicine, Samsung Medical Center, Sungkyunkwan University School of Medicine, Seoul, South Korea; e Department of Medicine, Faculty of Medicine, Siriraj Hospital, Mahidol University, Bangkok, Thailand; f St Lukes Medical Centre, Quezon City, Philippines; g Department of Medicine, University of Malaya, Malaysia; h Institute of Haematology and Blood Transfusion, Vietnam; i Department of Medicine, Hong Kong University and Hong Kong Sanatorium & Hospital, Hong Kong; j National Taiwan University Hospital, Taipei, Taiwan; k Beijing Chao-Yang Hospital and Capital Medical University, China; l Division of Hematology and the Division of Hematopathology, Mayo Clinic, Rochester, MN, USA; m Cedars-Sinai Medical Center, Los Angeles, CA, USA; n National University Cancer Institute and National University Hospital, Singapore; o Kawashima Hospital, Tokushima University, Japan

**Keywords:** Plasma cell disorders, Multiple myeloma, Bone disease, Bone imaging, Smouldering myeloma

## Abstract

The role of imaging in accurate classification and management of plasma cell disorders is substantial with the increasing evidence in the ability of cross-sectional imaging to identify disease related manifestations. International myeloma working group and nation-wide guidelines provide recommendations for guiding practice. However, there are remarkable variations in practice globally and in particular, adoption of incorrect imaging techniques due to lack of awareness/education, lack of equipment or personnel and resource constraints. These limitations are not specific to any particular geographic area. Hence in this complex and evolving imaging landscape, clinicians require practical guidance incorporating up-to-date and emerging evidence that is relevant to their healthcare system. In this manuscript we describe the two-pronged (minimum and enhanced) recommendations for imaging in different categories of plasma cell disorders from the Asian myeloma network bone imaging workgroup arrived through a questionnaire-based inquiry of prevailing imaging practices, discussion of those practices in the light of optimal evidence-based imaging recommendations, barriers in adoption of those recommendations and suggestions to overcome some of these barriers. There is an important role for these regionally relevant but globally applicable recommendations rooted in clinical practice which can serve as a medium for Physician/nursing, patient/carer education and to enable dialogue with healthcare technology appraisers/funders and secure interventions required to optimise practice in this field.

**Search strategy and selection criteria**

Primary references for the development of consensus were identified by discussions within the AMN Work Group co-chairs and members, with a focus on widely accepted international guidelines on the topic, and additional ones selected by topic group presenters and discussants. All references were discussed, disclosed, and distributed prior to the consensus discussions by the work group co-chairs. Further reference inputs for inclusion if any were sought from the IMWG bone imaging guidance authors. Only papers published in English were considered. The final reference list was generated on the basis of originality and relevance to the consensus statements.

## Introduction

Bone lesions are a highly prevalent and debilitating disease-defining feature of multiple myeloma (MM) with more than 80% of patients developing them during their disease course.^1^ These skeletal related events are associated with higher mortality,^2^ worse quality of life,^3^ and healthcare costs.^4^ Imaging of bone disease plays an important role in overall management of plasma cell disorders, from diagnosis and prognostication to identification of complications and treatment response assessment. Early identification of skeletal involvement could lead to changes in therapy and disease management.

The landscape of imaging for myeloma bone disease is evolving with the advent of novel cross-sectional imaging techniques and protocols, including whole-body low-dose CT (WBLDCT), whole-body MRI (WBMRI) and 18F-FDG PET/CT (PET/CT). Skeletal survey with plain X-rays (Conventional Skeletal Survey/CSS) which was the standard of care imaging technique in the past, has been shown to be inferior in detecting early bone lesions particularly in spine and pelvis and tends to underestimate the presence of active disease (in up to 40% of cases) when compared with PET/CT^5^ or WBLDCT.^6^^,^^7^ Focal lesions detected on MRI and PET, as specified in the international myeloma working group (IMWG) criteria, help not only to assess the structural integrity of bone by detecting early lesions affecting the mineralised bone before cortical destruction has occurred but also detect areas of focal bone marrow conversion caused by aggregates of plasma cells^8^; the latter is particularly important since myeloma bone marrow involvement is often patchy. Hence these assessments are fundamentally different from detecting lytic lesions on conventional skeletal survey where the bone destruction has already taken place—when patients are perhaps symptomatic with bone pains and are already experiencing consequences of catastrophic fractures. Early detection of the lesions during the course of myeloma is paramount as surgical, radiotherapeutic or mechanical interventions prior to the occurrence of fractures can help to minimise pain and complications, reduce disability, preserve quality of life, and reduce healthcare costs associated with rehabilitation. In addition, there are further added advantages like early detection of second primary malignancies or asymptomatic localised abscesses/infections when using PET/CT. Judicious use of PET/CT, WBMRI, and WBLDCT are associated with minimal harm as they do not require contrast agents, and the latter is performed at lower radiation dose compared to conventional CT.^9^

The IMWG has published consensus guidelines on the optimal use of imaging methods for the detection of bone and bone marrow lesions in the management of plasma cell disorders. However, various challenges exist in the standardisation of imaging practice according to the best-practice guidelines across countries and regions in Asia. Asian Myeloma Network (AMN) was established by the international myeloma foundation and is comprised of myeloma experts from China, Hong Kong, Malaysia, Taiwan, Japan, Philippines, Singapore, South Korea, Vietnam, and Thailand. Since its founding, the AMN has established the epidemiology of myeloma in Asian countries, as a basis for region-specific treatment management tools and strategies, launched and overseen clinical trials, enabled myeloma patients to gain access to the newer myeloma treatments, developed informational resource materials in local languages, especially for patients and caregivers and engaged in collaborative studies. Hence, with the growing importance of imaging in MM, the Asian Myeloma Network (AMN) undertook a questionnaire-based study to investigate the prevailing practices and propose a working consensus to guide evidence based clinical practice in the region.

The objectives of this study were: (1) To document and evaluate the imaging practices in plasma cell disorders across AMN member countries and regions in Asia with reference to IMWG consensus recommendations and (2) to identify factors which influence the decision-making regarding the choice of diagnostic imaging modalities with a view to explore solutions and overcome barriers.

## Consensus process

Members of the Asian Myeloma Network (panel of Haematologists with clinical and research interest in multiple myeloma) who were participants of the bone imaging workgroup at the 6th AMN summit between 14 and 16th Oct 2022, discussed institutional and national practices in relation to aspects of bone imaging in various categories of plasma cell neoplasms as well as barriers to compliance with IMWG recommendations. A bone imaging survey questionnaire was designed from the inputs collected during this summit and the pre-summit workgroup (conducted on the 9th of Sep 2022). This was circulated to the wider Asian Myeloma Network members between 16th Oct 2022 and 30 Nov 2022. The participants spanned countries and regions across Asia including China, Japan, South Korea, Malaysia, Taiwan, Thailand, Philippines, Vietnam, and Singapore from public and private hospitals in tertiary as well as peripheral or district setting [Figure S1] and were all Haematologists with an interest in management of plasma cell disorders.

In the questionnaire [Figure S2], participants were asked to indicate their preferred first-line imaging choice for categories of plasma cell disorders. They were asked to rank in order of preference of their imaging practice–CSS, WBLDCT, WBMRI, and 18F-FDG PET/CT for five clinical plasma cell disorder scenarios–suspected myeloma (to differentiate MGUS/SMM Vs Symptomatic MM), confirmed newly diagnosed myeloma (when there was already an indication to treat), solitary plasmacytoma, myeloma treatment response assessment, and diagnosis of extra-medullary disease. Participants were also asked to indicate barriers to establishing cross-sectional imaging service for myeloma at their respective institutions. A sample questionnaire is shown in Figure S2. The results were then collated and analysed.

The collated survey results were presented to the members of the bone imaging work group who convened for the pre-summit workgroup and deliberated on the results during the 7th AMN summit held in Bangkok on 20th–22nd Oct 2023. Prevailing practices relating to imaging of each of the plasma cell disorder categories as gleaned from the survey were discussed—current standard of care practices, aspects relating to how they compared or deviated from the recommended IMWG guidance, reasons for such occurrence, practice improvement opportunities, barriers and measures for consideration to potentially overcoming these barriers. These were used to generate a consensus recommendation for imaging practice that was appropriate and relevant for the region. In addition, to accommodate for the differences that exist within the different care settings these recommendations were classified as minimally appropriate and enhanced standard based on best available evidence [Table 1].

**Table 1:** Summary of current practice, consensus recommendations, and measures required to meet consensus recommendations.

	Current practice	Minimum recommendation	Enhanced standard/IMWG recommendation	Issues to consider	Initiatives/measures/support for practice change
First line imaging in suspected MM	25% centres using CSS as 1st line imaging Other centres using PET/CT/WBMRI/MRI spine and pelvis	Move from CSS to cross sectional (Xs) imaging (WBLDCT) Choice of Xs imaging based on local reimbursement/availability	PET CT—1st line imaging If PET is Neg or inconclusive then WBMRI CSS not recommended	CSS leads to underdiagnosis of symptomatic MM and leads to undertreatment that can prevent catastrophic organ damage Inability of differentiate SMM from symptomatic MM by IMW criteria	In centres with CT scanners—switch to WBLDCT Guidance on WBLDCT protocols Education of physicians/patients/nurses Discussion with regulators for funding support
Bone disease screening in NDMM	15% centres using CSS as 1st line imaging Other centres using PET/CT. WBMRI. MRI spine and pelvis–used in few	Move from CSS to cross sectional (Xs) imaging (WBLDCT) Choice of Xs imaging based on local reimbursement/availability	WBDLCT PET CT—1st line imaging If Neg or inconclusive WBMRI	CSS leads to underdiagnosis of asymptomatic bone lesions which can lead to fractures 12% false neg rates with PET CT Standardisation of acquisition and reporting of diff imaging modalities.	Guidance on WBLDCT protocols as above Funding to run standardised PET CT service Education of physicians/patients/nurses
Response assessment in treated myeloma	55% participants used PET CT 10% used whole-body MRI WBDLCT (19%) 15% participants reported using skeletal survey At least 10% participants do not routinely perform imaging	PET-CT should be used if response assessment is being performed and a baseline is available (and was positive for lesions) If no prior PET-CT, use the same imaging for comparison and if no initial scan was performed—WBLDCT is the choice CSS is NOT recommended	PET-CT—1st line imaging Diffusion-weighted whole-body MRI can be an alternative when FDG-PET/CT cannot be performed/or the baseline was MRI Response assessment is for prognostic importance ONLY presently—no change in treatment recommended UNLESS there is there is PD by IMWG criteria	PET-CT can give false-negative and/or false-positive results. When patient is in serological response for PET-avid lesions comparison of CT components is advised as it provides information on bone healing (residual lesions can take time to resolve)	Avoid skeletal survey Funding to run standardised PET CT and whole-body MRI service Prospective data collection is encouraged—opportunity to consider an AMN wide study Local reimbursement/availability Education of physicians/patients/nurses
Plasmacytoma	78% centres using PET-CT as 1st line imaging WBMRI and WBLDCT are used in 11% and 17%, respectively	PET/CT as preferred modality due to access and availability and for comparability with follow-up imaging WBMRI or spine and pelvis MRI as alternative	WBMRI 1st choice (Spine and Pelvis MRI if WBMRI is not feasible) PET CT close alternative Repeat same technique for follow-up imaging	WBLDCT—not suggested since role unclear CSS—no role Follow-up imaging	Funding to run standardised PET CT service Education of physicians/patients/nurses
Extramedullary disease	72% centres using PET-CT as 1st line imaging Other centres use PET/CT + WBMRI (4%), WBMRI (6%), WBLDCT (4%), and all of these (13%). Skeletal survey is not selected	PET/CT is recommended	PET-CT—1st line imaging	Local reimbursement/availability PET-CT can give false-negative and/or false-positive results.	Funding to run standardised PET CT service Local reimbursement/availability

## Results

### Survey results and consensus recommendations

48 responses were received from 37 public tertiary or academic hospitals, 9 from public district or peripheral hospitals, and 2 from private healthcare institutions. The prevailing practices as reported via the responses under the five categories (suspected myeloma, confirmed myeloma, plasmacytoma, extramedullary disease, treatment response assessment), the IMWG guidance for the respective categories and the consensus for each of those categories are presented below.

### Suspected myeloma

Suspected myeloma in this context refers to patients with a monoclonal paraprotein without confirmed myeloma or an indication to treat, i.e., patients with monoclonal gammopathy of uncertain significance, smouldering myeloma, or asymptomatic myeloma.

Incidence of precursor plasma cell disorders in Asia is less well documented but is generally thought to be less common than in the west.^10^, ^11^, ^12^ However, the incidence and prevalence of myeloma in Asia has been increasing over the last two decades and while the reasons for this could be many, including changes in lifestyle, occupational and environmental exposures, and food intake among other factors, it could also be due to differences in access to health care, diagnostic modalities, data collection methods, and other influences unrelated to the natural history or evolution of plasma cell disorders. Radiological imaging plays a crucial role in the accurate classification of plasma cell disorders based on the updated IMWG definitions. For accurate classification and delineation of patients into MGUS/SMM/asymptomatic and symptomatic myeloma, the IMWG guidelines recommends the use of WBLDCT as the first-line imaging of choice with PET/CT and WBMRI as second line imaging; the latter is recommended particularly when suspicion is high and WBLDCT is negative or inconclusive.

In our survey, the preferred imaging modalities for screening bone disease in patients suspected to have myeloma were split between skeletal survey (n = 13, 27%), whole-body low dose CT (n = 13, 27%), and PET/CT (n = 14, 29%) [Figure S3]. Hence, while two-thirds of the respondents chose WBLDCT/PET/CT or WBMRI as first line imaging there were 27% of respondents still employing conventional skeletal survey as their first-line modality. Skeletal survey is less sensitive in picking up radiological osteolytic lesions compared to CT and using CSS leads to underdiagnoses of lytic lesions in up to 25% of patients.^6^ Since the incorrect classification of patients in this category of suspected myeloma has great potential for harm, a shift away from CSS to WBLDCT is essential. This was agreed to be the most practical and readily adoptable recommendation for the region. However, a significant barrier exists in the form of non-availability of WBLDCT protocols even in tertiary centres with contemporary CT scanning equipment. Protocols for generating WBLDCT images by incorporating appropriate software techniques into prevailing CT scanning equipment have been published and are readily available. Discussion between haematologists and radiologists to this aspect and exploring the feasibility of adoption of WBLDCT protocols can help many centres to switch from CSS to WBLDCT for screening. To this end, these protocols were shared within the group to allow for adoption of WBLDCT protocols. Appeal to health care funding departments to make provision for such equipment or personnel with experience to man such equipment will also be important to provide such service. In addition, education for physicians, nurses, and patients can play a huge role in creating awareness for the need and for adoption of cross-sectional imaging in this in-need group of patients. In centres where there is access and expertise as was the case in 37% of our respondents' institutions, PET/CT or MRI can be used as the imaging of choice or used as modality to clarify suspicious lesions on CT.

One of the challenges of employing the optimal imaging modality is the inability to predict the clinical phenotype of any given patient's disease manifestations. For example, while a patient might progress with clinically significant bone lesions some might have increased marrow accumulation of plasma cells. It should be noted that in the former scenario a WBLDCT or PET CT might be the ideal modality for earlier detection but in the latter, it would be best detected early by a WBMRI. Hence an inherent difficulty in the choice of ideal imaging modality needs to be appreciated. This could potentially result in a patient having more than one type of cross-sectional imaging which can lead to escalating costs. Hence as much as possible the optimal modality needs to be carefully chosen in a given patient's clinical context.

### Confirmed newly diagnosed myeloma

Imaging prior to treatment initiation in patients who have confirmed symptomatic myeloma has prognostic importance.^13^, ^14^, ^15^ Number, extent, and response of lesions to myeloma treatment have been shown to be an independent predictor of survival.^16^ Baseline and repeat imaging assists in monitoring for disease evolution or relapse which can prompt initiation or change in treatment.

In our survey, for patients with newly diagnosed symptomatic myeloma based on non-bone related myeloma-defining event, 98% of participants indicated that they would still routinely screen for bone disease. We thought that financial constraints might lead physicians to omit bone imaging altogether for this group of patients, however, the survey confirmed otherwise. The importance of this good practice cannot be emphasised enough. Early identification of asymptomatic areas of bone disease can lead to early bone directed treatment as well as monitoring and provides an opportunity for early intervention including prophylactic surgical interventions that can minimise catastrophic fractures/associated mortality/morbidity including hospitalisation, infections, reduced QOL, and productivity.^17^ The preferred mode for 1st line imaging in patients with confirmed newly diagnosed myeloma was PET/CT (n = 25, 52%) however almost half of the participants opted for other modalities including skeletal survey (n = 7, 15%), whole-body low-dose CT (n = 10, 21%), and whole-body MRI (n = 6, 13%). All except 1 participant (n = 47, 98%) answered that they would routinely screen myeloma patients for bone disease even if the patient had another myeloma-defining event warranting treatment.

The advantages of screening and identification of bone disease in a newly diagnosed myeloma patient are evident. Just as in the precursor conditions, the IMWG guideline recommends the use of WBLDCT as the first-line imaging of choice with WBMRI as second line imaging, when the suspicion is high and WBLDCT is negative or inconclusive. A significant number of institutions were still employing conventional skeletal survey. Given the substantial evidence in support of the superiority of cross-sectional imaging, the need for concerted efforts to move away from CSS was unanimous at the consensus generating discussions. From a patient perspective, CSS can be challenging for those with bone pain or poor mobility due to the long duration of assessment and body positioning required to obtain radiographic images. Therefore, WBLDCT is the preferred technique recommended as a minimal requirement that should be employed in this group of patients.

PET/CT is recommended as an alternative to WBMRI, and can be used as a sole imaging modality at diagnosis and for serial monitoring. It was apparent that majority of physicians from the region preferred PET/CT over MRI due to cost, practical difficulties of arranging stepwise imaging (WBLDCT followed by WBMRI as indicated in guidelines) and lack of availability of WBLDCT or WBMRI in some countries/regions. One study from UK looked at prospective comparison of WBMRI and FDG PET CT in 60 patients and reported a slightly better ability of MRI in picking up skeletal lesions that PET CT. Given that the difference in pick-up rate between the modalities was noted in only 2 patients at a level where based on current definitions diagnostic classification would not have changed, the significance of this remains unknown.^18^

Where PET/CT is employed, it must be recognised that false-negative FDG PET may be detected in up to 12% of patients at diagnosis and during the course of the disease, because of a lack of the hexokinase enzyme.^19^ It is however expected that the CT component of the examination would still diagnose lytic lesions even if the PET component is negative. Furthermore, it is recommended where possible, to replace the CT of a PET/CT for mere attenuation correction with a CT that has diagnostic image quality. Additionally, though the utility of PET/CT in prognosis is clear with documented evidence in the Asian population, the timing of the examinations, consensus on significant baseline/residual SUV values and the value of it in determining minimal residual disease are all evolving. As such change in management based on these findings is discouraged outside a clinical trial context. Adoption of standardised recommendations^7^^,^^20^^,^^21^ for acquisition, interpretation, and reporting of all imaging techniques while training and harmonisation of PET/CT reporting for myeloma in line with the updated evidence is encouraged.

### Plasmacytoma

Plasmacytoma is divided into the following types by IMWG: solitary plasmacytoma (SP) of bone, extramedullary plasmacytoma, and multiple plasmacytomas. These disease entities are characterised by clonal plasma cell tumour(s) with little or no M-protein in serum and urine and without bone marrow infiltration consistent with multiple myeloma. Solitary plasmacytoma is regarded as a precursor or an early manifestation of MM; therefore, plasma cell infiltration in the bone marrow should be thoroughly evaluated to exclude systemic clonal plasma cell infiltration, and follow-up of any lesion(s) already detected is also mandatory during the course of the disease. The same technique used at initial diagnosis should be repeated.

To diagnose and classify these types of plasma cell disorders accurately full skeletal imaging with a whole-body MRI and PET-CT are crucial in anyone with suspected solitary plasmacytoma^22^ to exclude additional osteolytic lesions or soft tissue masses which would constitute symptomatic multiple myeloma.^23^^,^^24^ Whole body MRI and PET/CT provide the ability to detect diffuse infiltration and focal bone marrow infiltration.^25^ Among the imaging modalities, MRI is generally accepted to be the most sensitive and specific for the detection of myeloma infiltration in the bone marrow, which can lead to the early detection of a focal or diffuse myeloma infiltration in the bone marrow before the appearance of osteolytic lesions.^8^ Therefore, significant numbers of patients with apparently solitary plasmacytoma have been reported to be upstaged to multiple myeloma with MRI.^21^^,^^26^ IMWG recommends whole-body MRI in patients with newly diagnosed solitary bone plasmacytoma or MRI of the spine and pelvis if whole-body MRI is not available.^27^ If MRI is not available, PET-CT can be used as an alternative in patients with newly diagnosed solitary bone plasmacytoma. For extramedullary lesions, including solitary extramedullary plasmacytoma, PET-CT is the preferred imaging modality to detect and exclude further lesions.

In our survey, for plasmacytoma, PET/CT was the preferred first-line imaging modality (n = 32, 78%) followed by WBLDCT (n = 8, 17%), and whole-body MRI (n = 5, 11%) [Figure S5]. Hence most centres were compliant to the suggested imaging guidance. WBLDCT is not a modality of choice generally for this group of patients since there is a need to pick up soft tissue lesions as well and the resolution for that is much superior with MRI and PET/CT. Hence education of the physicians to this aspect of the approach to imaging in plasmacytoma could be one measure to improve practice. If WBMRI is not accessible due to cost or logistics, either PET/CT or a Spine/Pelvis MRI could be alternatives. In the survey, distinction between bone-based SP versus extramedullary SP was not made. It should be recognised that MRI would be preferred in the former whilst PET/CT would be preferred in the latter clinical situation.

### Extramedullary disease in MM patients

Patients with multiple myeloma may develop extramedullary lesions at diagnosis and/or during the course of the disease, especially in the later stages. Whole-body imaging is needed to pick up extramedullary disease. PET/CT is an excellent modality to identify extra medullary disease^28^, ^29^, ^30^, ^31^ and is the imaging of choice for detecting extramedullary disease per the IMWG guidance due to its sensitivity.^27^ In our survey, PET/CT was preferred as the first-line imaging choice for identifying extramedullary disease (n = 34, 72%); participants also utilised PET/CT plus whole-body MRI (4%), whole-body MRI alone (6%), whole-body low-dose CT (4%), and all of these (13%) [Figure S6]. Except for a small (4%) proportion of centres (who were using WBLDCT), it was apparent that most were using/able to use the appropriate modality for this group of patients. Education and exploring barriers to adopting PET CT as discussed below are to be considered for optimising practice for this aspect of plasma cell disorder management.

### Treatment response assessment

The response of MM to treatment is firstly evaluated by serum and urine tests and a bone marrow aspirate. However, bone marrow assessments from one site of the pelvic bone alone cannot reflect tumour response in other sites of bone marrow and outside of bone in the body, given the spatial heterogeneity characteristic of MM. As patients with multiple myeloma may develop extramedullary lesions and the distribution of lesions in the bone marrow is not uniform, whole-body imaging studies are necessary to determine treatment efficacy and monitor the disease course. Owing to the introduction of novel agents into therapeutic strategies, the outlook for myeloma patients has greatly improved over the past decade. And now, high quality response with minimal residual disease negativity can be attained and serve as a surrogate for survival and has become a treatment goal in and outside of clinical trials. When patients achieve CR with negative minimal residual disease, the current accepted gold standard of whole-body imaging for response assessment of MM is PET-CT.^31^ For patients with residual lesions detected by PET/CT, yearly follow-up is recommended. Diffusion-weighted whole-body MRI can be an alternative when PET/CT cannot be performed. During follow-up, generally, the same imaging modality performed at baseline should be used to allow comparison.

Results of our survey on the first line imaging choice for routine response assessment are provided [Figure S7]. More than half of participants used PET/CT (n = 23, 55%) for this purpose. Whole-body MRI can be an alternative to PET-CT, and was selected by about 10% of the participants. Remaining participants (about 40%) opted for other modalities including skeletal survey (15%) and whole-body low-dose CT (19%). At least 5 participants shared that they do not routinely perform imaging for disease response assessment (for example, following autologous stem cell transplant or induction therapy in transplant ineligible patients).

CSS is generally inappropriate for response assessment and should be avoided to limit radiation exposure. Since most centres preferred PET/CT as initial investigation, it is the most appropriate modality for response assessment since same imaging technique used at diagnosis has the advantage of comparability. When performed as a planned imaging during one phase of treatment (before maintenance for example), it has to be recognised that PET avidity alone should not be taken as sign of non-response or progression and that residual PET avid lesions (especially when in serological response) can with longer follow-up become negative. Furthermore, correlating with the CT component might be useful in such situations as that might show evidence of sclerotic healing. For centres performing WBMRI it wasn't clear from the survey whether diffusion weighted imaging was available and used. If available DW WBMRI could be preferred alternative in the absence of PET/CT.

### Barriers and potential solutions

The most common barrier to establishing cross-sectional imaging like WBLDCT, WBMRI, and PET/CT identified from the survey were limitation in resources, particularly financial restrictions or lack of reimbursement reported by 60% (n = 28) of participants. 81% of participants reported financial reimbursements for skeletal survey, only about half of participants reported subsidisation for WBMRI (56%) or PET/CT (54%). WBLDCT was subsidised for 71% (n = 34).

Resource limitation also included limited access to advanced imaging technology with 13% (n = 6) reporting lack of scanning equipment and 8.5% (n = 4) reporting lack of scanning personnel. 3 participants reported lack of available nuclear medicine specialist or radiologist capable of interpreting the necessary imaging protocols.

Whilst healthcare funding and investment into improving diagnostics or the access to it or improving manpower capability could be a solution to address some of the disparities in practice this wouldn't be a readily feasible universal solution. Simple adoption of WBLDCT protocols by disseminating and educating the know-hows of standardisation of CT protocols for optimising to bone imaging in myeloma could see a significant improvement without a major expenditure especially in centres which already possess the CT scanning equipment and radiologists. A bone imaging focussed training for involved specialists was felt as a useful solution by many centres and is being planned. One of the key consensus findings was to have a regional guideline with recommendations of imaging which would enable discussions with stakeholders from the centres to the health care funders and health technology appraisers. Finally, the role of education in optimisation of bone imaging cannot be overemphasised.

Education of all the stakeholders—physicians, nurses, patients, radiologists, and others involved in the care of myeloma patients is a hugely important component in trying to address the barriers involved.

## Additional considerations

In our survey, WBLDCT was generally preferred over WBMRI in most clinical scenarios. The benefits of WBLDCT include the speed, tolerability, and the relatively low dose of radiation—the newer reconstruction techniques allow for radiation doses only slightly higher than that required for skeletal survey.^32^ WBLDCT is outperforming CSS even in difficult to assess areas such as the skull and ribs^33^ and has added value in operative planning to assess spinal stability and vertebral fractures. Low-dose CT offers a balance between image quality and a lower radiation dose, while still providing detailed images for early detection of pathological changes in soft tissues in addition to bone disease. Whole-body low dose CT should be included in routine work-up tests for diagnosis of multiple myeloma.

One study comparing the diagnostic performance of WBLDCT to WBMRI in myeloma suggested that MRI had higher detection rate and improved staging for patients with myeloma (n = 41), (11 patients upstaged with MRI compared with CT).^34^ A standard WBMRI protocol includes a T1 weighted and diffusion-weighted sequence from the vertex to the knees,^20^ with consideration of gadolinium enhancement to increase sensitivity for those without renal impairment. MRI is also superior for evaluation of soft tissue involvement or spinal cord assessment. On the downside, the average duration of WBMRI scan is 45 min which may be an issue for hospitals with incumbered scanner capacity already.^21^

All whole-body imaging should include the vertex of the skull to at least the knees. If protocols are available, the lower extremities should be displayed in full. IMWG cautions against overinterpretation, recommending that suspicious lesions smaller than 5 mm should be re-imaged with CT or MRI in 3–6 months.^27^ It should be noted that FDG-PET/CT can give false-negative results when the expression of hexokinase-2 in myeloma cells is low or absent, and false-positive results with use of G-CSF and pathological conditions, including inflammation and fractures. FDG-PET/CT may not be suitable for determining treatment efficacy in such cases. Pros and cons for whole-body imaging modalities are provided in Table 2.

**Table 2:** Pros and cons of whole-body imaging modalities.

	WBLDCT	WBMRI	18FDG/PET
Pros	• Low radiation • Wide availability • Short acquisition time • Assess spinal stability and osteolytic lesions • Ability to detect sclerotic healing in fracture/lytic lesional sites post-treatment	• No radiation • Detects early non-lytic infiltration with the highest sensitivity • Prognostic significance of number of focal lesions and diffuse pattern • Extraosseous assessment • Superior assessment of spinal cord/nerve compression • Assessment of therapy response using DWI	• Detection of lytic lesions and areas of focal marrow plasma cell infiltration • Prognostic significance and ability to detect minimal residual disease • Highly sensitive for extra-medullary disease • Ability to detect second primary malignancy/focal infected areas
Cons	• Cannot detect marrow infiltration/conversion • Cannot differentiate active Vs inactive treated disease • Limited assessment of spinal • Cord/radicular compression • Limited assessment of soft tissues plasmacytomas	• High cost • Not widely available • Long examination time	• Higher radiation • Higher cost • Limited availability in secondary centres • Limited assessment of spinal cord/radicular compression • Need to consider false-positive and negative results

Although whole-body MRI and PET/CT are currently recommended in detecting myeloma lesions, a number of practical issues remain barriers to deploy whole-body MRI and PET/CT in Asian countries/regions. Among others, shortage of community hospitals equipped with more expensive modalities such as whole-body MRI and PET/CT is critical. Other issues include 1) inadequate reimbursements that hamper the widespread adoption of these modalities; 2) a shortage of radiologists who can adequately interpret radiological images in whole-body modalities; and 3) standardisation in the skills of radiographers. Nonetheless, use of PET/CT and/or whole-body MRI should be justified in patients with NDMM in Asian countries.

When whole body imaging is reported, the pathology and image characteristics of fatty marrow in the elderly and concomitant osteoporosis should be also taken into account. Differentiating between diffuse myeloma lesions and rarefied trabeculae in patients with osteoporosis can be difficult. Dedicated training to radiographers and radiologists may be required at some centres for successful implementation of the protocol. Joint works by haematology and radiology societies in various regions might help to guide better use of whole-body imaging modalities. Patient education is also important to raise awareness of whole-body imaging modalities as the first line imaging choice without CSS to determine bone disease and bone marrow infiltration in patients with myeloma. Recommendation statements like ours based on current situations in Asian countries for haematologists, radiologists, and radiographers would be helpful to establish and standardise the practice among Asian countries/regions. Within the region, in Hong Kong for example, there has been experience in using alternative tagged isotopes (C11Ac) in place of FDG which appears to enhance the sensitivity of picking bone lesions which are negative with FDG due to use of alternative metabolic pathways^35^ other than glycolysis in MM cells. These haven't been explored in our survey or guidelines.

Our survey results need to be interpreted in context as this was conducted within the framework of an academic myeloma community—and hence it may not be representative of the practices of the whole region, as there may be local variations in practice that are not reflected in our results. However, the use of sub-optimal imaging like skeletal survey at key time points during the management of plasma cell disorders underlines the importance of standardisation.

## Conclusion

There is considerable variation in imaging practices in myeloma with variable adherence to evidence based IMWG recommendations in centres across Asia. While there has been a shift towards usage of cross-sectioning imaging over skeletal survey, there are likely to be non-clinical aspects that influence diagnostic imaging choices.

Resource limitation, specifically lack of financial subsidies for imaging, infrastructure or lack of experienced personnel all remain as major challenges in the implementation of standardised imaging practice for myeloma in Asia. However increased adoption of cross-sectional imaging at least in large tertiary centres are to be welcomed and suggest that in these centres, practices do closely mirror those in the recommendations. Increasing awareness of the potentially catastrophic nature of myeloma bone disease and the preventive role that cross sectional imaging could provide, dialogues with healthcare funders regarding this aspect of myeloma care, incorporations of low dose CT protocols to help move away from skeletal survey in centres which already have CT scanners could all be useful tools to enhance bone imaging standards in Asia. The role of education to enable better patient and physician understanding of the role of advanced imaging techniques in accomplishing the above is paramount. Patient societies and support groups meetings can be a valuable opportunity to share and disseminate the information.

Focused regional guidelines for imaging practice,^36^ will be helpful to encourage a shift in the standard of care and provide justification for increased resource investment in the necessary imaging services at institutions across Asia. One key motivation for this consensus recommendation that is rooted in clinical practice improvement, is to seek, enable a dialogue and work with the funders or regulatory/health technology appraisers/national societies tasked with establishing guidelines to secure the required interventions that would facilitate the practice standardisation based on best available evidence. The patients within the region can benefit hugely from the adoption of advanced cross-sectional imaging in the management of plasma cell malignancies.

## Contributors

JH, CN designed the questionnaire; CN and MA led the workgroups and consensus discussions.

CN, NFA, MA collated the responses and wrote the initial manuscript; all authors contributed to questionnaire, participated in pre-summit and in-summit workgroups, consensus discussions, critically reviewed the manuscript.

## Declaration of interests

JH declares grants from Bristol Myers Squibb and Glaxo Smith Kline and honoraria from Glaxo Smith Kline, Amgen, Janssen, Beigene, Angitia, Prothena, Sebia, Regeneron as well as association with committees in International myeloma working group and alliance for clinical trials.

MA declares receiving honoraria from Janssen, Takeda, Daiichi Sankyo, Sanofi, Bristol Myers Squibb and participation in DSMB/Advisory boards for Chugai Pharmaceutical and Bristol Myers Squibb.

KHK declares receiving grants from J & K, BMS, Amgen, Takeda, and Sanofi; consulting fees from J & K, BMS, Amgen, Takeda, Sanofi, Abbvie, and GSK; participation in DSMB/Advisory boards for J & K, BMS, Amgen, Takeda, Sanofi, Abbvie, and GSK.

Rest of the authors declare no relevant COI for this manuscript.

## Appendix A Supplementary data

Figures S1–S7

Survey on Myeloma Bone Imaging in AMN

Questionnaire Respondents
