# Supplementary material for: Imaging in plasma cell disorders—consensus recommendations of the Asian myeloma network bone imaging workgroup
Source: Lancet Reg Health West Pac. 2025 Jun 7;59:101597. doi: 10.1016/j.lanwpc.2025.101597 (PMC12174565; doi:10.1016/j.lanwpc.2025.101597)
Supplement: Figures S1–S7 [file mmc1.docx]

Figures

**Figure S1 – AMN Members participating in Survey on Myeloma Imaging Practices**

**Figure S2 – Link to Questionnaire in supplementary material**

**Figure S3 – Preferences for screening bone disease in patients with suspected myeloma**


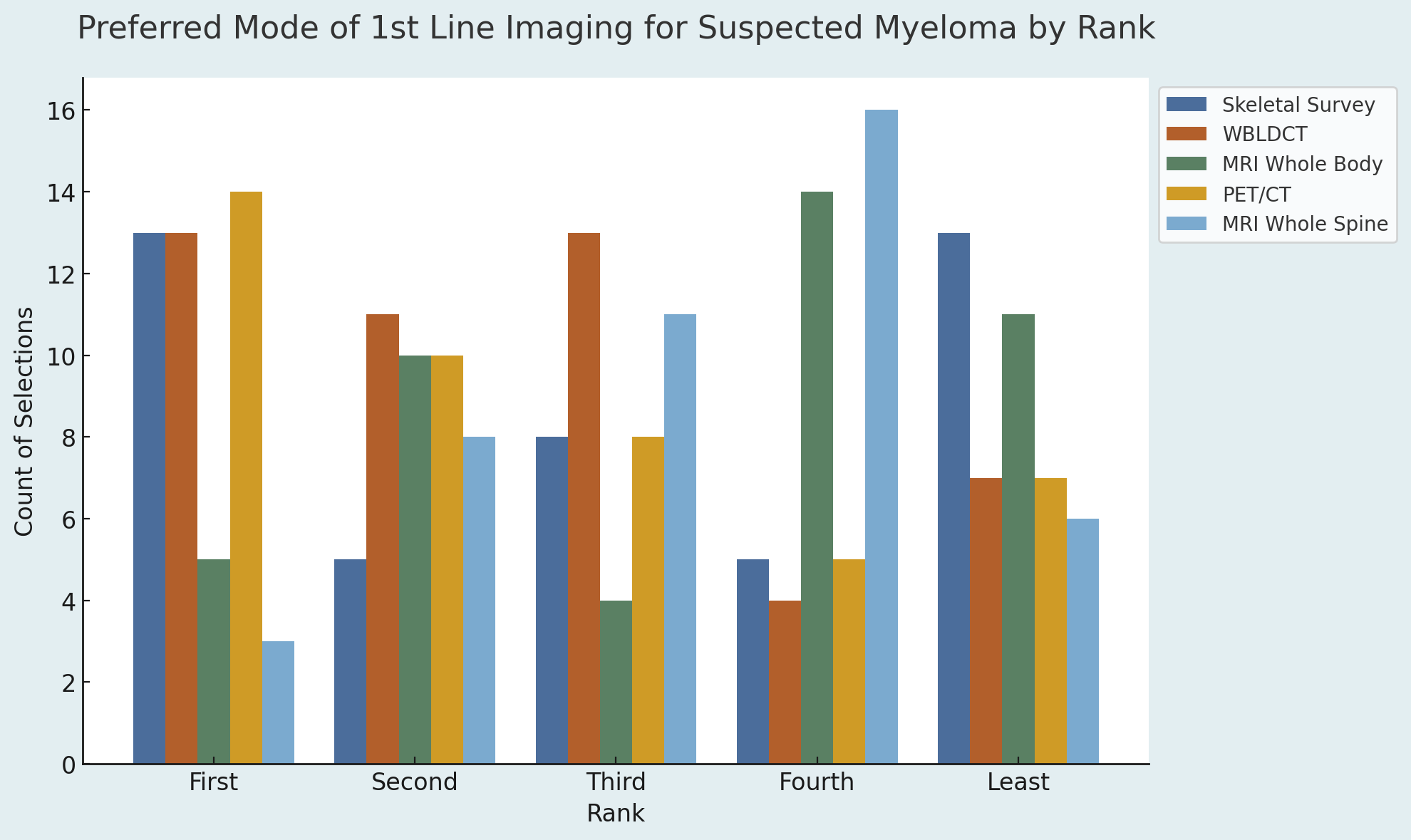


Ranking preferred choice of imaging in patients with suspected myeloma from most preferred (first) to least preferred (least). CSS conventional skeletal survey, WBLDCT whole body low dose CT

**Figure S4 – Preferences for evaluating bone disease in patients with confirmed myeloma**


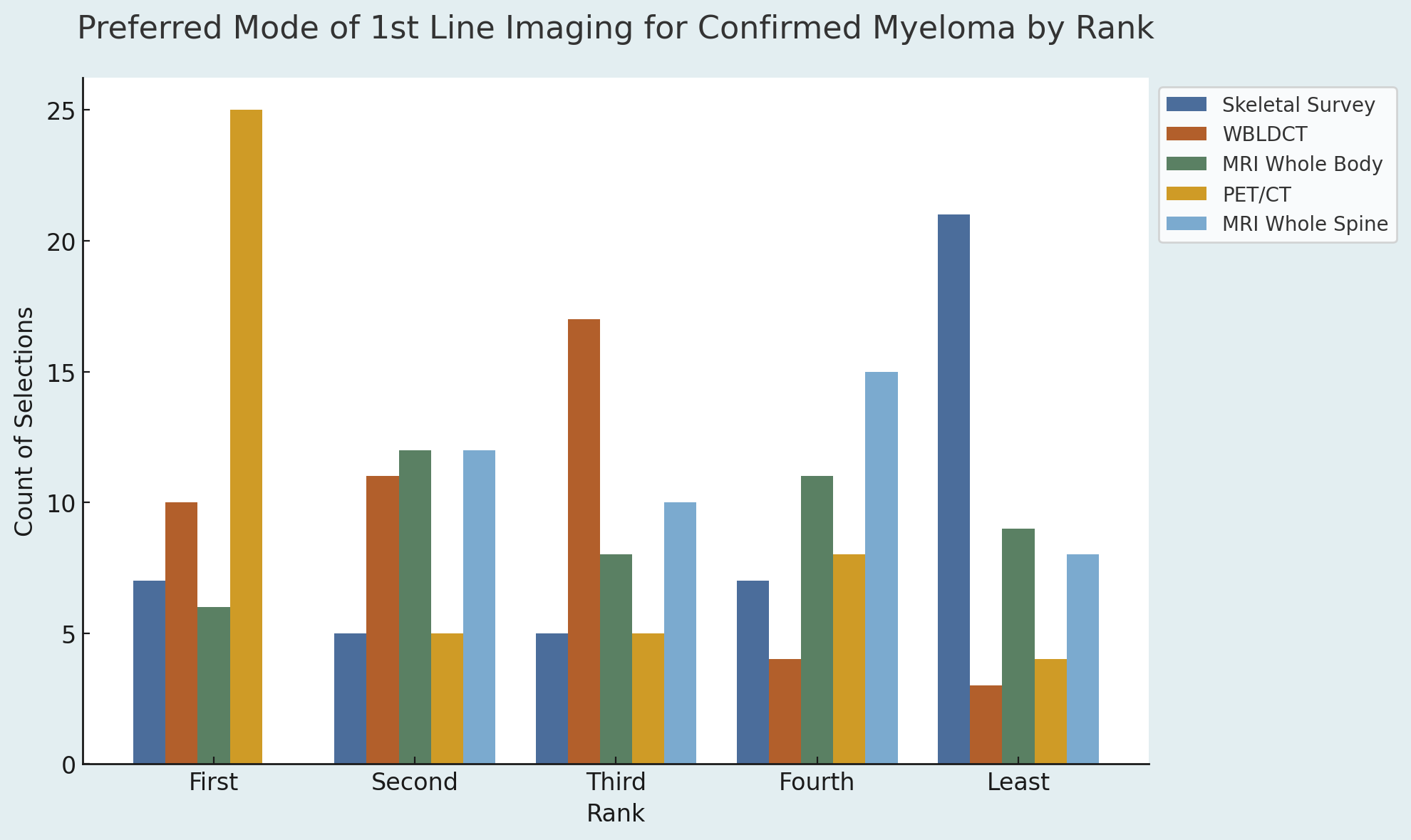


Ranking preferred choice of imaging in patients with confirmed myeloma from most preferred (first) to least preferred (least) CSS conventional skeletal survey, WBLDCT whole body low dose CT

**Figure S5 -** **Preferences for evaluating bone disease in patients plasmacytoma**


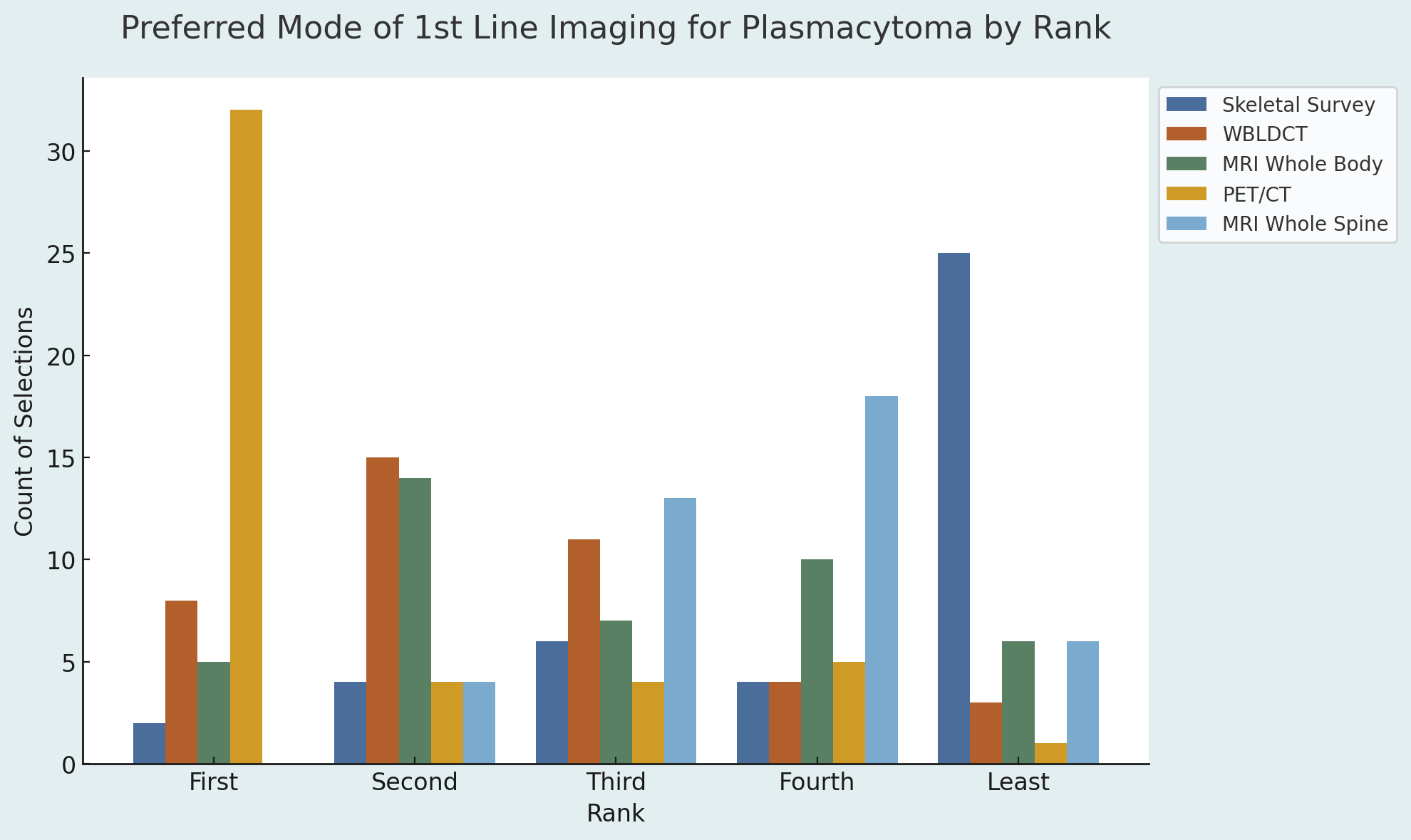


Ranking preferred choice of imaging in patients with plasmacytoma from most preferred (first) to least preferred (least)

CSS conventional skeletal survey, WBLDCT whole body low dose CT

**Figure S6 - Preferences for evaluating bone disease in patients with extramedullary disease**

**Figure S7 – Preferences for imaging for routine response assessment in myeloma**
