# Supplementary material for: Imaging in plasma cell disorders—consensus recommendations of the Asian myeloma network bone imaging workgroup
Source: Lancet Reg Health West Pac. 2025 Jun 7;59:101597. doi: 10.1016/j.lanwpc.2025.101597 (PMC12174565; doi:10.1016/j.lanwpc.2025.101597)
Supplement: Survey on Myeloma Bone Imaging in AMN [file mmc2.pdf]

Survey on Myeloma Bone Imaging in AMN centres

You are invited to participate in this 5-minute survey on the approach to bone-imaging in patients with suspected and/or confirmed plasma cell disorders including myeloma within the Asian Myeloma Network Centres. Though the International Myeloma Working Group recently established guidelines on optimal use of imaging methods at different disease stages (J Hillengass et al Lancet Oncol 2019; 20: e302–12) there are considerable variations in practice geographically and even between centres in a country. We believe it is important to understand these variations, why they exist, so that they can inform future resource planning, allocation and lead to standardized evidence-based detection and management practices across the AMN. This survey is supported by International Myeloma Foundation / Asian Myeloma Network and results of this will be discussed during the proceedings of the Asian Myeloma Network Summit at Singapore in Oct 2022.

* Indicates required question

1. Email *

____

2. **Name of the Institution:** *

____

3. **Country:** *

____

4. **Type of Institution** *

Check all that apply.

- [ ] Public Healthcare – Tertiary referral / University academic centre and or Specialist Myeloma centre
- [ ] Public Healthcare – District general / Peripheral centre
- [ ] Private Hospital / Health care Institution

5. Please rank your preferred mode of 1st line imaging, used for screening bone disease in patients ***suspected*** to have Myeloma

(From 1 to 5; 1 being the most preferred and 5 the least preferred)

Mark only one oval per row.

	1	2	3	4	5
**Skeletal Survey**					
**Whole Body Low Dose CT**					
**Whole Body MRI**					
**FDG labelled PET CT**					
**MRI Whole Spine**					

6. In your practice, do you routinely screen newly diagnosed myeloma patients for bone disease if they have another Myeloma defining event requiring treatment? *

Mark only one oval.

- Yes
- No
- Other: __________

7. Please rank your preferred mode of 1st line bone imaging in patients with confirmed ***newly diagnosed*** myeloma.

(From 1 to 5; 1 being the most preferred and 5 the least preferred)

Mark only one oval per row.

	1	2	3	4	5
**Skeletal Survey**	◯	◯	◯	◯	◯
**Whole Body Low Dose CT**	◯	◯	◯	◯	◯
**Whole Body MRI**	◯	◯	◯	◯	◯
**FDG labelled PET CT**	◯	◯	◯	◯	◯
**MRI Whole Spine**	◯	◯	◯	◯	◯

8. What are the imaging modalities that are subsidized / re-imbursed in your country for newly diagnosed myeloma patients? (Tick all that apply)

Check all that apply.

- [ ] Skeletal Survey
- [ ] Whole Body Low Dose CT
- [ ] Whole Body MRI
- [ ] FDG labelled PET CT
- [ ] MRI Whole Spine
- [ ] Other: ____________

9. Following from previous question, are there conditions for re-imbursement of the imaging modality?

Mark only one oval.

- Yes – applicable only for baseline screening
- Yes – applicable only if symptoms
- No – can use for screening and response assessment
- Other: ______________

10. Please rank your preferred mode of bone imaging for routine response assessment in NDMM patients - for example 3/12 post ASCT or end of induction therapy in Transplant Ineligible patients?
(From 1 to 5; 1 being the most preferred and 5 the least preferred)

Mark only one oval per row.

	1	2	3	4	5
**Skeletal Survey**					
**Whole Body Low Dose CT**					
**Whole Body MRI**					
**FDG labelled PET CT**					
**MRI Whole Spine**					

11. Please rank your preferred mode of 1st line imaging in patients with ***Plasmacytoma***

(From 1 to 5; 1 being the most preferred and 5 the least preferred)

Mark only one oval per row.

	1	2	3	4	5
**Skeletal Survey**	⬭	⬭	⬭	⬭	⬭
**Whole Body Low Dose CT**	⬭	⬭	⬭	⬭	⬭
**Whole Body MRI**	⬭	⬭	⬭	⬭	⬭
**FDG labelled PET CT**	⬭	⬭	⬭	⬭	⬭
**MRI Whole Spine**	⬭	⬭	⬭	⬭	⬭

12. What is your imaging modality of choice for identifying extramedullary disease?

Mark only one oval.

- ⬭ Whole Body Low Dose CT
- ⬭ Whole Body MRI
- ⬭ FDG labelled PET CT
- ⬭ Other: ______________________________

13. What barriers do you need to overcome to establish a PET – CT / MRI service for Myeloma at your centre?

Check all that apply.

- [ ] Not applicable as my centre already has such a service
- [ ] Scanning equipment availability
- [ ] Scanning personnel availability or training
- [ ] Availability of reporting Radiologists or Nuclear medicine specialist
- [ ] Financial / Reimbursement / Subsidy
- [ ] Other: ______________________

14. Please provide any additional information that you would want to tell us regarding myeloma bone imaging at your center if not covered in the above questions.

____

____

____

____

____

This content is neither created nor endorsed by Google.

Google Forms
