# Supplementary material for: Imaging in plasma cell disorders—consensus recommendations of the Asian myeloma network bone imaging workgroup
Source: Lancet Reg Health West Pac. 2025 Jun 7;59:101597. doi: 10.1016/j.lanwpc.2025.101597 (PMC12174565; doi:10.1016/j.lanwpc.2025.101597)
Supplement: Questionnaire Respondents [file mmc3.pdf]

Participants who responded to questionnaire and their affiliations		
1.	Shanghai Renji Hospital, China	Dr. Jian Hou
2.	The affiliated hospital of inner Mongolia medical university, China	Dr. Da Gao
3.	Shenjing hospital of China Medical University, China	Dr. Aijun Liao
4.	Beijing Chao-Yang Hospital, China	Dr. Wenming Chen , Dr. Wen Gao
5.	Qilu Hospital Shandong University, China	Dr. Luqun Wang
6.	Jiangsu Province Hospital, China	Dr. Lijuan Chen, Dr Xiaoyan Qu
7.	Shanghai Jiaotong University Medical School affiliated Ruijin Hospital, China	Dr. Jianqin Mi
8.	Peking Union Medical College Hospital, China	Dr. Junlin Zhuang
9.	The first affiliated hospital of Sun Yat-sen University, China	Dr. Juan Li
10.	Changzheng Hospital, Naval Medical University, China	Dr. Juan Du
11.	West China Hospital, China	Dr. Li Zhang
12.	Fujian Medical University Union Hospital, China	Dr. Rong Zhan
13.	First affiliated hospital of Soochow Universit, China	Dr. Chengcheng FU
14.	The second hospital of shanxi medical university, China	Dr. Aili He
15.	Qingdao Municipal Hospital, China	Dr. Yuping Zhong
16.	Chinese academy of Medical sciences, China	Dr. Lugui Qiu
17.	Hong Kong University and Gleneagles Hospital	Dr. Chim Chor Sang James
18.	Nagoya University, Japan	Dr. Kazuyuki Shimizhu
19.	Kyoto prefectural University, Japan	Dr. Kuro Jun
20.	Iwate Medical University, Japan	Dr. Shigeki Ito
21.	Dokkyo Medical University, Japan	Dr. Yoichi Imai
22.	Tokushima Prefectural Central Hospital, Japan	Dr. Shuji Ozaki
23.	The University of Osaka Graduate School of Medicine, Japan	Dr. Naoki Hosen
24.	NHO Hiroshimanishi Medical Center, Japan	Dr. Yoshiaki Kuroda
25.	Japanese Red Cross Medical Center, Japan	Dr. Tadao Ishida
26.	Ogaki Municipal Hospital, Japan	Dr. Hiroshi Kosugi
27.	Kawashima Hospital, Tokushima University, Japan	Dr. Masahiro Abe
28.	Kanazawa University, Japan	Dr. Hiroyuki Takamatsu
29.	Samsung Medical centre, Korea	Dr. Kihyun Kim
30.	Yonsei University College of Medicine, Severance Hospital	Dr. Jin Seok Kim
31.	Asan Medical Center, University of Ulsan College of Medicine, Korea	Dr. Dok Hyun Yoon
32.		
33.	St Lukes Medical Centre, Quezon City, Philippines	Dr. Jay Datukan
34.	Chiang Mai University, Thailand	Dr. Ekarat Rattarittamrong
35.	Siriraj Hospital, Mahidol University, Thailand	Dr. Chutima Kunacheewa
36.	Ramathibodhi Hospital, Thailand	Dr. Teeraya Puavilai
37.	Singapore General Hospital	Dr. Chen Yunxin
38.	Singapore General Hospital	Dr. Chandramouli Nagarajan
39.	Tan Tock Seng Hospital, Singapore	Dr. Allison Tso
40.	Mount Elizabeth Hospital, Singapore	Dr. Daryl Tan
41.	National University Hospital, Singapore	Dr. Chng Wee Joo
42.	Hospital Ampang, Malaysia	Dr. Sen Mui Tan
43.	University of Malaya, Malaysia	Dr. Gin Gin Gan
44.	Subang Jaya Medical centre, Malaysia	Dr. Ng Soo Chin

45.	Queen Elizabeth Hospital, Sabah, Malaysia	Dr. Khaiteri Ragunathan
46.	Chan Bing Show Chwan Memorial Hospital, Taiwan	Dr. Cheng-Shyong Chang
47.	National Taiwan University Hospital, Taiwan	Dr. Jeffery Huang
48.	Institute of Haematology and Blood transfusion, Vietnam	Dr. Vo Thi Thanh Binh
